# Efficacy and Safety of Brinzolamide as Add-On to Prostaglandin Analogues or β-Blocker for Glaucoma and Ocular Hypertension: A Systematic Review and Meta-Analysis

**DOI:** 10.3389/fphar.2019.00679

**Published:** 2019-06-25

**Authors:** Yuanzhi Liu, Junyi Zhao, Xiaoyan Zhong, Qiming Wei, Yilan Huang

**Affiliations:** ^1^Department of Pharmacy, The Affiliated Hospital of Southwest Medical University, Luzhou, China; ^2^College of Pharmacy, Southwest Medical University, Luzhou, China

**Keywords:** brinzolamide, prostaglandin analogues, β-blocker, glaucoma, ocular hypertension, systematic review

## Abstract

**Background:** Brinzolamide as a carbonic anhydrase inhibitor could be combined with other intraocular pressure (IOP) lowering drugs for glaucoma and ocular hypertension (OHT), but the efficacy was controversial. So, this study was used to assess the efficacy and safety of brinzolamide as add-on to prostaglandin analogues (PGAs) or β-blocker in treating patients with glaucoma or OHT who fail to adequately control IOP.

**Methods:** We searched PubMed, Embase, MEDLINE, Cochrane Library, and clinicaltrials.gov from inception to October 4, 2018. Randomized controlled trials of brinzolamide as add-on to PGAs or β-blocker for glaucoma and OHT were included. Meta-analysis was conducted by RevMan 5.3 software.

**Results:** A total of 26 trials including 5,583 patients were analyzed. Brinzolamide produced absolute reductions of IOP as an adjunctive therapy for patients with glaucoma or OHT. Brinzolamide and timolol were not significantly different in lowering IOP as add-on to PGAs (9 am: *P* = 0.07; 12 am: *P* = 0.66; 4 pm: *P* = 0.66). Likewise, brinzolamide was as effective as dorzolamide in depressing IOP (9 am: *P* = 0.59; 12 am: *P* = 0.94; 4 pm: *P* = 0.95). For the mean diurnal IOP at the end of treatment duration, there were no statistical differences in above comparisons (*P* > 0.05). Compared with brimonidine (b.i.d.), there was a significant reduction of IOP in brinzolamide (b.i.d.) at 9 am (*P* < 0.0001); however, the difference was cloudy in thrice daily subgroup (*P* = 0.44); at 12 am, brinzolamide (b.i.d.) was similar to brimonidine (b.i.d.) in IOP-lowering effect (*P* = 0.23), whereas brimonidine (t.i.d.) led to a greater effect than brinzolamide (t.i.d.) (*P* = 0.02). At 4 pm, brinzolamide (b.i.d.) was superior IOP-lowering effect compared with brimonidine (b.i.d.) (*P* = 0.0003); conversely, the effect in brinzolamide (t.i.d.) was lower than brimonidine (t.i.d.) (*P* < 0.0001). For the mean diurnal IOP, brinzolamide was lower in twice daily subgroup (*P* < 0.00001); brimonidine was lower in thrice daily subgroup (*P* < 0.00001). With regard to the safety, brinzolamide and dorzolamide had a higher incidence of taste abnormality; moreover, brinzolamide resulted in more frequent blurred vision; dorzolamide resulted in more frequent ocular discomfort and eye pain. Timolol resulted in more frequent blurred vision and less conjunctival hyperemia. Brimonidine resulted in more frequent ocular hyperemia. As to other adverse events (AEs) (conjunctivitis, eye pruritus, foreign body sensation in eyes, and treatment-related AEs), brinzolamide was similar to other three active comparators.

**Conclusions:** Brinzolamide, as add-on to PGAs or β-blocker, significantly decreased IOP of patients with refractory glaucoma or OHT and the AEs were tolerable.

## Introduction

Glaucoma is an acquired disease of irreversible blindness and the second leading cause of blindness worldwide, characterized by optic neuropathies and intraocular pressure (IOP) elevation (Peters et al., 2014). Primary open-angle glaucoma (POAG), one of the most prevalent types, will have threatened 76.0 million people by 2020 and 111.8 million people by 2040 (Tham et al., 2014). There are no significant symptoms in the early stage of glaucoma, but once showing impaired vision, the patients have lost nearly 1 million of their retinal ganglion cell (RGCS) (Weinreb et al., 2014; Sharif, 2018). Therefore, the early diagnosis and treatment are particularly important for glaucoma.

Currently, pharmacotherapy is still a common and effective way to treat glaucoma and ocular hypertension (OHT). There are a variety of IOP-lowering agents containing carbonic anhydrase inhibitors (CAIs), beta-blockers, α2-adrenergic agonists, and prostaglandin analogues (PGAs). PGAs are the first-line treatment option, while their monotherapies may offer insufficient IOP control, so they need to be combined with other therapies, such as latanoprost and travoprost, which are combined with brinzolamide or dorzolamide or brimonidine or timolol for patients failing to control IOP (Cheng et al., 2009; Dzhumataeva, 2016; Lusthaus and Goldberg, 2017). Timolol, one of the β-blockers, has an obvious effect on diurnal IOP, but it is also insufficient to hold a stable IOP over the long term (Konstas et al., 2016). Brimonidine, an α2-adrenergic agonist, is popularized due to the positive effect of AQH and neuroprotective actions (Lusthaus and Goldberg, 2017). Brinzolamide and dorzolamide could inhibit carbonic anhydrase in ciliary epithelium to reduce IOP, increase retinal blood flow, and the efficacy of brinzolamide would be enhanced after improving the drug-delivery system (Iester, 2008; Konstas et al., 2013; Dong et al., 2018; Wang et al., 2018). However, brinzolamide and dorzolamide are restricted by lacking efficacy and brimonidine has a higher AE. It is, therefore, essential to combine multiple agents.

According to the differences of mechanisms, brinzolamide could be used in combination with other IOP-lowering drugs for glaucoma and OHT. However, there were no relevant systematic reviews to compare the efficacy and safety between brinzolamide and other active drugs as add-on treatment. Thus, basing on published and unpublished randomized controlled trials (RCTs) of patients with glaucoma or OHT, we did a systematic review to assess the efficacy and safety of brinzolamide compared with other anti-glaucoma agents as add-on treatment.

## Methods

This systematic review was conducted in accordance with the Preferred Reporting Items for Systematic Reviews and Meta-Analyses (PRISMA) statement (Knobloch et al., 2011).

### Data Sources and Search Strategy

We systematically searched using databases including PubMed, Embase, MEDLINE, and Cochrane Library from inception to September 4, 2018, with a language restriction (English). The unpublished data were also searched from clinicaltrials.gov. We used the following terms: “brinzolamide,” “CAS No. 138890-62-7,” “carbonic anhydrase inhibitors (CAI),” “glaucoma,” and “ocular hypertension.” These terms were adjusted to adhere to the relevant rules in each database.

Two independent reviewers screened titles and abstracts of all retrieved citations, and subsequently examined potentially eligible studies in full text. All discrepancies were resolved through discussion and added to the third reviewer when necessary.

### Study Selection and Data Extraction

We included RCTs if they met the following criteria: 1) patients aged > 18 years; 2) a clinical diagnosis of glaucoma (POAG, exfoliation glaucoma, pigmentary glaucoma) or OHT in at least one eye (study eye); 3) the patients without lowering IOP adequately by the monotherapies of antiglaucomatous drugs (PGA: IOP ≥ 18 mmHg; β-blocker: IOP ≥ 20 mmHg) or the patients with IOP ≥ 20 mmHg without medication (including washout schedule); 4) the patients using brinzolamide as a monotherapy or a combination therapy for safety analysis; 5) no history of glaucoma surgery before the study; 6) Snellen visual acuity ≥ 0.1 or Snellen score ≥ 20/100 in the study eye(s); 7) duration: follow-up time ≥ 4 weeks; and 8) outcome variables: a) IOP changes from baseline; b) the mean diurnal IOP at the end of treatment duration; c) AEs.

Exclusion criteria were as follows: 1) a history of chronic or recurrent severe ocular inflammatory disease; 2) ocular trauma or intraocular surgery within 6 months or laser eye surgery within 3 months of screening; 3) ocular infection, endophthalmitis, or retinal disease; 4) hypersensitivity to any of the excipients in the study medications; 5) maximum corrected visual acuity ≤ 0.2 (decimal acuity) or an anterior chamber angle grade < 2 in either eye; 6) quantify visual acuity < 0.6 logarithm of the minimal angle resolution; 7) optic nerve with a cup-disc ratio > 0.8; 8) previous or current evidence of a severe illness or any other condition that could make the patient unsuitable for the study; 9) treatment with stable doses of any medication within 30 days of the start of the study that could affect IOP; and 10) pregnant or lactating, or intending to become pregnant during the study period.

### Data Extraction and Risk of Bias Assessment

The data extraction was implemented by two independent reviewers (YL and QW) according to the inclusion criteria. The information extracted from the trials includes study characteristics, interventions, types of glaucoma, duration of treatment, background therapy, and efficacy outcomes and AEs.

The methodological quality of eligible studies was assessed using the Cochrane risk-of-bias tool (Higgins et al., 2011). The predefined key domains included: randomization, allocation concealment, blinding, intent-to-treat (ITT) analysis, and a description of losses to follow-up.

We chose doses of the study drugs including brinzolamide 1% b.i.d. or t.i.d., which were the most commonly used doses in clinical treatments. In addition, our studies included 23 articles published and 3 articles unpublished. All studies are assessed under the same criteria.

### Statistical Analysis

The statistical analysis was performed by 5.3 software and Stata 12 software. For the efficacy (IOP changes from baseline, the mean diurnal IOP at the end of treatment duration), we assessed them by the weighted mean difference (WMD) with 95% confidence intervals (CIs). For the safety, we assessed the incidences of AEs by risk ratios (RRs) with 95% CIs. Heterogeneity was evaluated with the chi-square test and the I^2^ statistic. We planned to explore heterogeneity with a sensitivity analysis when I^2^ was higher than 50% (Higgins et al., 2003). We also conducted egger analysis to assess the potential publication bias when three or more studies offered relevant data, and defined significant publication bias with the *P* value < 0.1.

## Results

### Search Results and Study Characteristics

**Figure 1:**
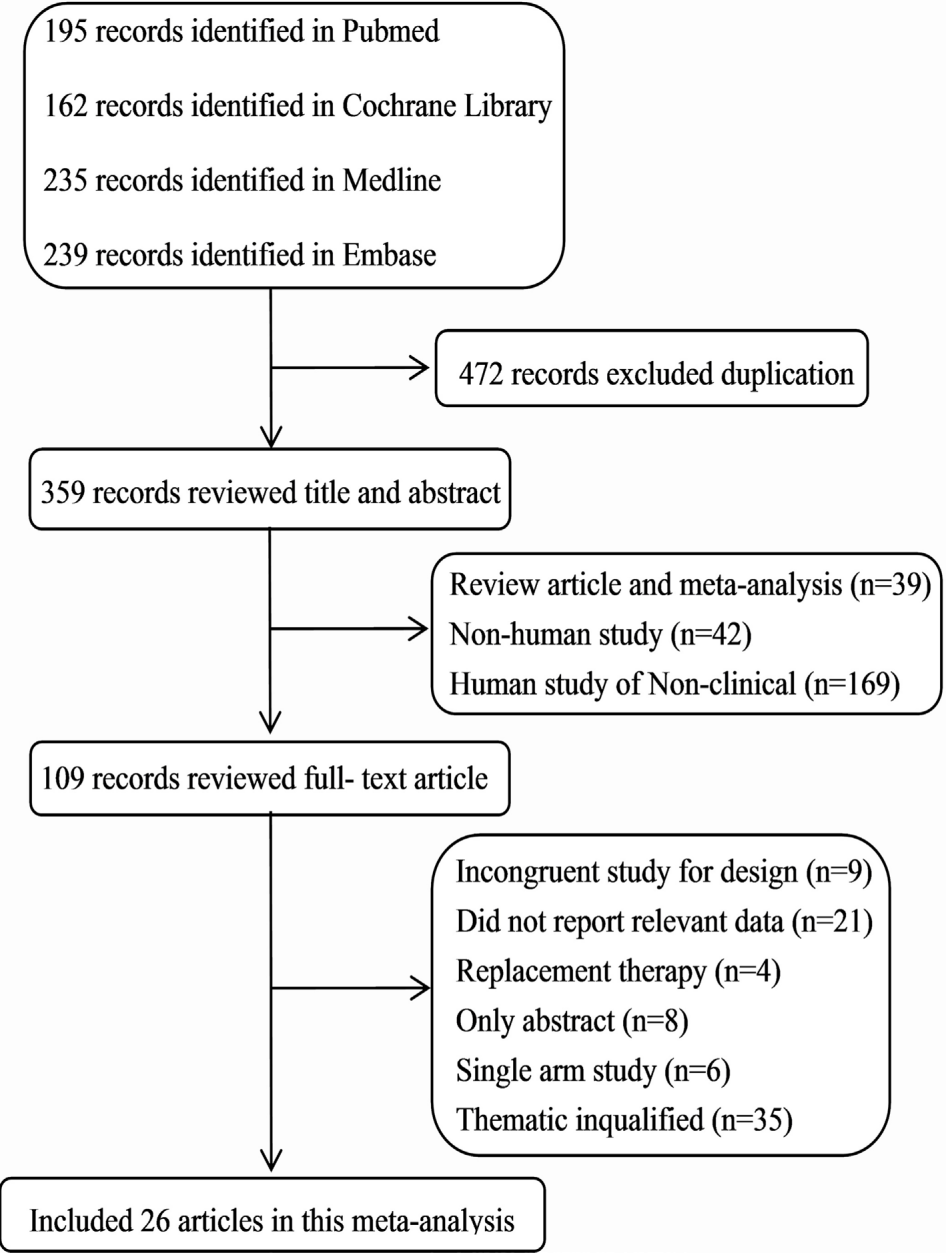
Flow chart of selected studies.

We identified 831 articles from four databases search through the search strategy, and 472 with duplicate were removed. After excluding reviews, meta-analysis, non-human studies, and non-clinical human studies, 109 were left. By further reviewing the full text, we included 26 articles with a total of 5,683 patients (**Figure 1**). The basic characteristics of the included studies were shown in** Supplementary Table 1**. All trials were randomized and active-controlled involving the study drugs added on PAG in 11 articles (Hollo et al., 2006; Reis et al., 2006; Feldman et al., 2007; Day and Hollander, 2008; Miura et al., 2008; Bournias and Lai, 2009; Nakamura et al., 2009; Pfeiffer, 2011; Konstas et al., 2013; Alcon, 2016; Aihara et al., 2017), added on timolol 0.5% in two articles (Michaud and Friren, 2001; Martinez and Sanchez-Salorio, 2009), and added on the combination therapy of latanoprost and a beta-blocker in one article (Tsukamoto et al., 2005). Main clinical diagnosis of patients were POAG and OHT; a few were other glaucoma (exfoliation glaucoma, pigmentary glaucoma). Duration of intervention ≥ 4 weeks.

### Bias Risk Analysis

**Supplementary Table 2** presented the bias risk analysis of the included RCTs. All studies were randomized, multicenter clinical trials; six trials (Silver, 1998; Miura et al., 2008; Bournias and Lai, 2009; Manni et al., 2009; Katz et al., 2013; Aung et al., 2014) described the sequence generation. Twelve studies (Silver, 1998; Sall, 2000; March and Ochsner, 2000; Hollo et al., 2006; Feldman et al., 2007; Day and Hollander, 2008; Kaback et al., 2008; Miura et al., 2008; Bournias and Lai, 2009; Pfeiffer, 2011; Katz et al., 2013; Aung et al., 2014) offer the details of concealment procedures (Martinez and Sanchez-Salorio, 2009; Research, 2013a; Research, 2013b; Alcon, 2016). Sixteen trials performed ITT analyses (Sall, 2000; March and Ochsner, 2000; Michaud and Friren, 2001; Hollo et al., 2006; Feldman et al., 2007; Kaback et al., 2008; Bournias and Lai, 2009; Martinez and Sanchez-Salorio, 2009; Manni et al., 2009; Pfeiffer, 2011; Research, 2013a; Research, 2013b; Katz et al., 2013; Nguyen et al., 2013; Whitson et al., 2013; Aung et al., 2014) and all studies described withdraws or dropouts. All studies were funded by the company.

### Efficacy Analysis

#### Brinzolamide vs Timolol

**Figure 2:**
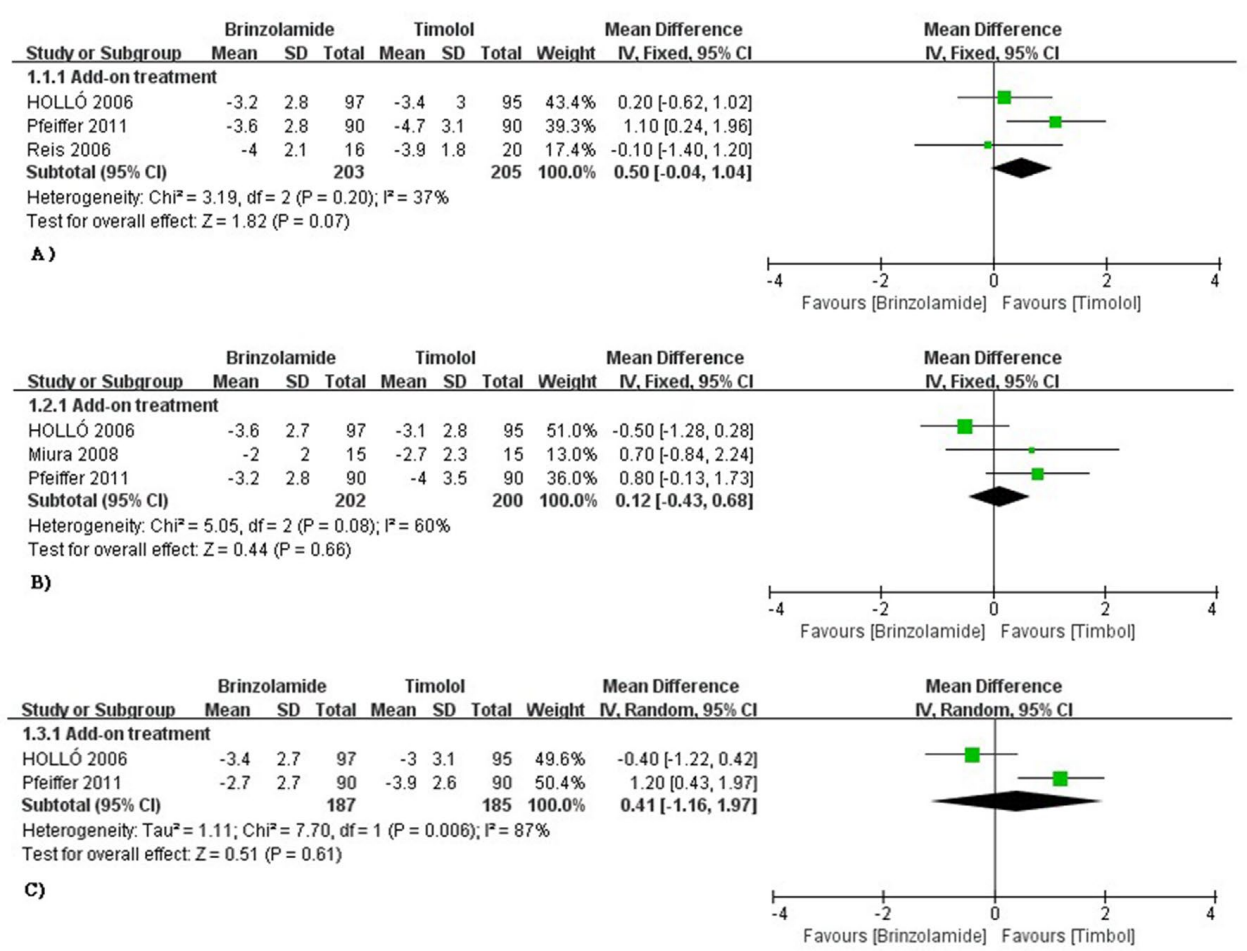
Forest plot for IOP change from baseline (Brinzolamide group vs. Timolol group). The plot of change value for IOP at 9 am **(A)**, 12 am **(B)**, 4 pm **(C)**.

**Figure 3:**
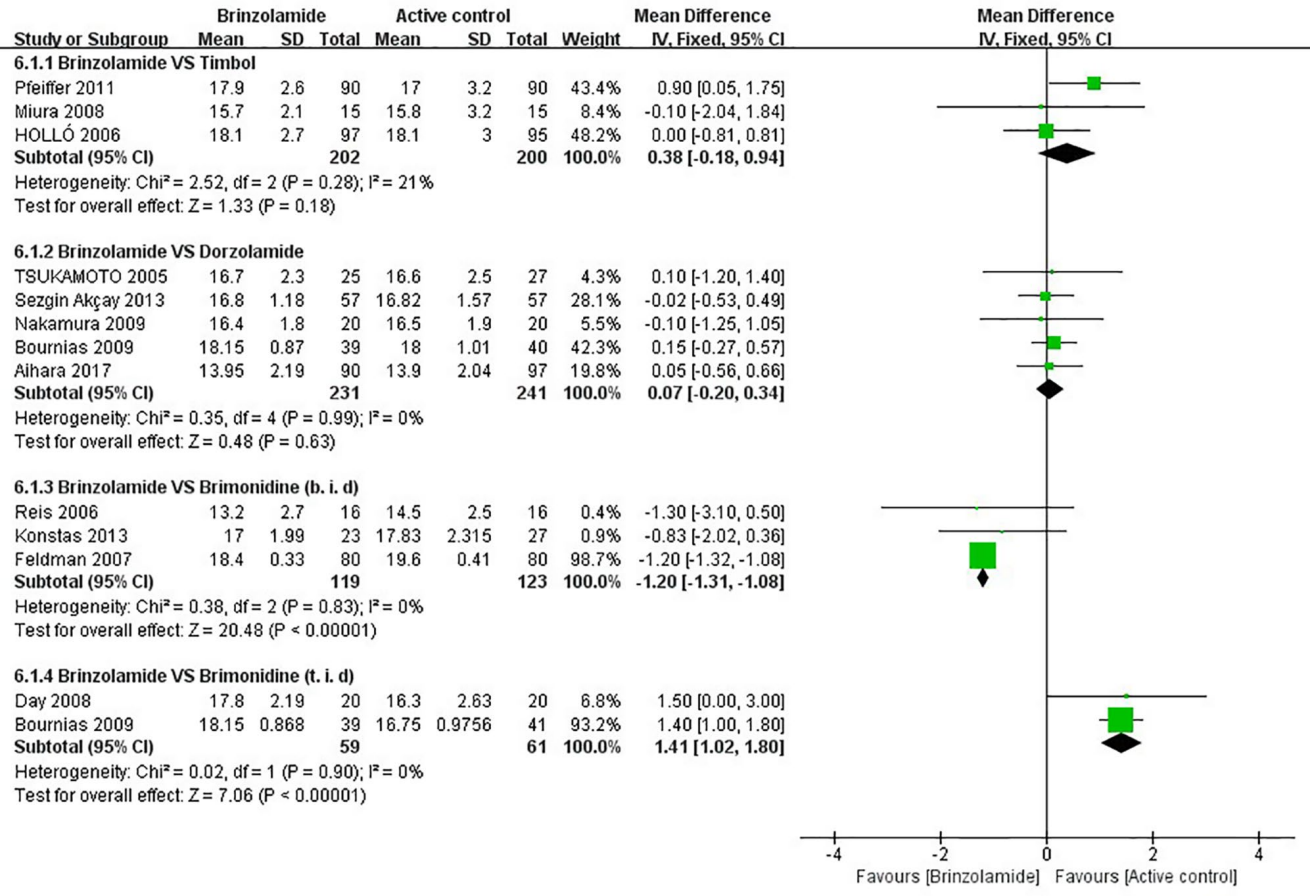
Forest plot for the mean diurnal IOP at the end of treatment duration (Brinzolamide group vs. active control group).The mean diurnal IOP between Brinzolamide group and Timolol group (6.1.1); between Brinzolamide group and Dozorlamide group (6.1.2); between Brinzolamide group and Brimonidine group (b.i.d) (6.1.3), (t.i.d) (6.1.4).

The changes of IOP from baseline between brinzolamide and timolol were shown in **Figure 2**. Both drugs significantly decreased IOP as adjunctive therapies to PGAs. There were no statistically significant differences (9 am: WMD 0.50 mmHg, 95%CI [−0.04 to 1.04], *P* = 0.07, *I* = 37%; 12 am: WMD 0.25 mmHg, 95%CI [−0.70 to 1.19], *P* = 0.61, *I* = 60%; 4 pm: WMD 0.41 mmHg, 95%CI [−1.16 to 1.97], *P* = 0.66, *I* = 87%). For the high level of heterogeneity at 12 am, we removed one trial (Hollo et al., 2006) whose designs slightly differ from others, and the heterogeneity was eliminated without affecting the overall estimate (WMD 0.77 mmHg, 95%CI [−0.02 to 1.57], *P* = 0.06, *I* = 0%). At 4 pm, we did not use a sensitivity analysis due to only including two trials. Likewise, the mean diurnal IOPs at the end of treatment duration did not differ between brinzolamide and timolol (WMD 0.38 mmHg, 95%CI [−0.18 to 0.94], *P* = 0.18, *I* = 21%) (**Figure 3**). There was no publication bias on egger test (*P* ≥ 0.1; **Supplementary Table 3**).

#### Brinzolamide vs Dorzolamide

**Figure 4:**
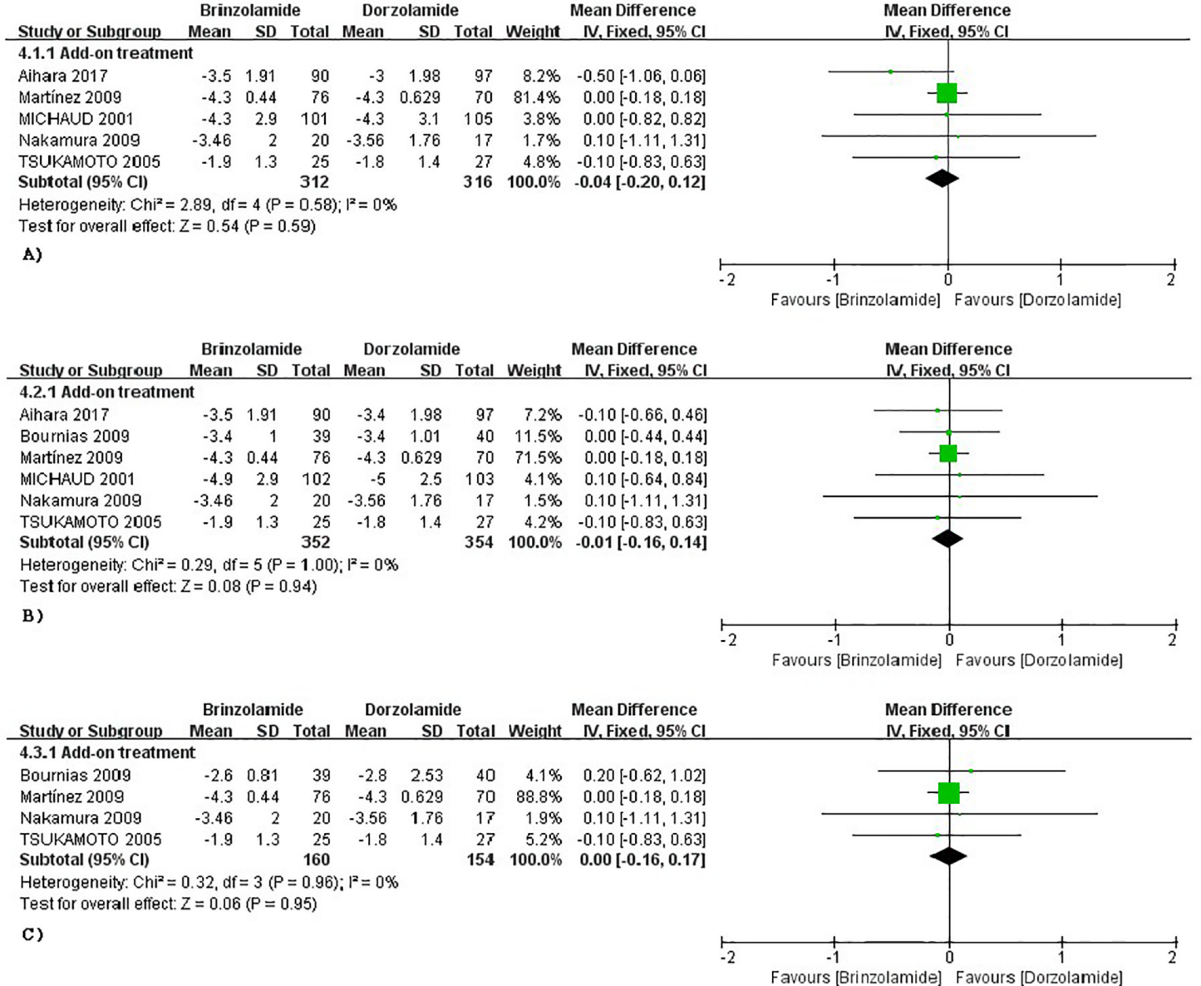
Forest plot for IOP change from baseline (Brinzolamide group vs. Dozorlamide group). The plot of change value for IOP at 9 am **(A)**, 12 am **(B)**, 4 pm **(C)**.

The changes of IOP from baseline between brinzolamide and dorzolamide were shown in **Figure 4**. Both drugs significantly decreased IOP as adjunctive therapies to PGAs and/or beta-blocker, and the brinzolamide was as effective as dorzolamide in depressing IOP (9 am: WMD −0.04 mmHg, 95%CI [−0.20 to 0.11], *P* = 0.59, *I* = 0%; 12 am: WMD −0.01 mmHg, 95%CI [−0.16 to 0.14], *P* = 0.94, *I* = 0%; 4 pm: WD 0 mmHg, 95%CI [−0.16 to 0.17], *P* = 0.95, *I* = 0%). The mean diurnal IOPs at the end were also similar (WMD 0.07 mmHg, 95%CI [−0.20 to 0.34], *P* = 0.63, *I* = 0%) (**Figure 3**). No publication bias on egger test was found (*P* ≥ 0.1; **Supplementary Table 3**).

#### Brinzolamide vs Brimonidine

**Figure 5:**
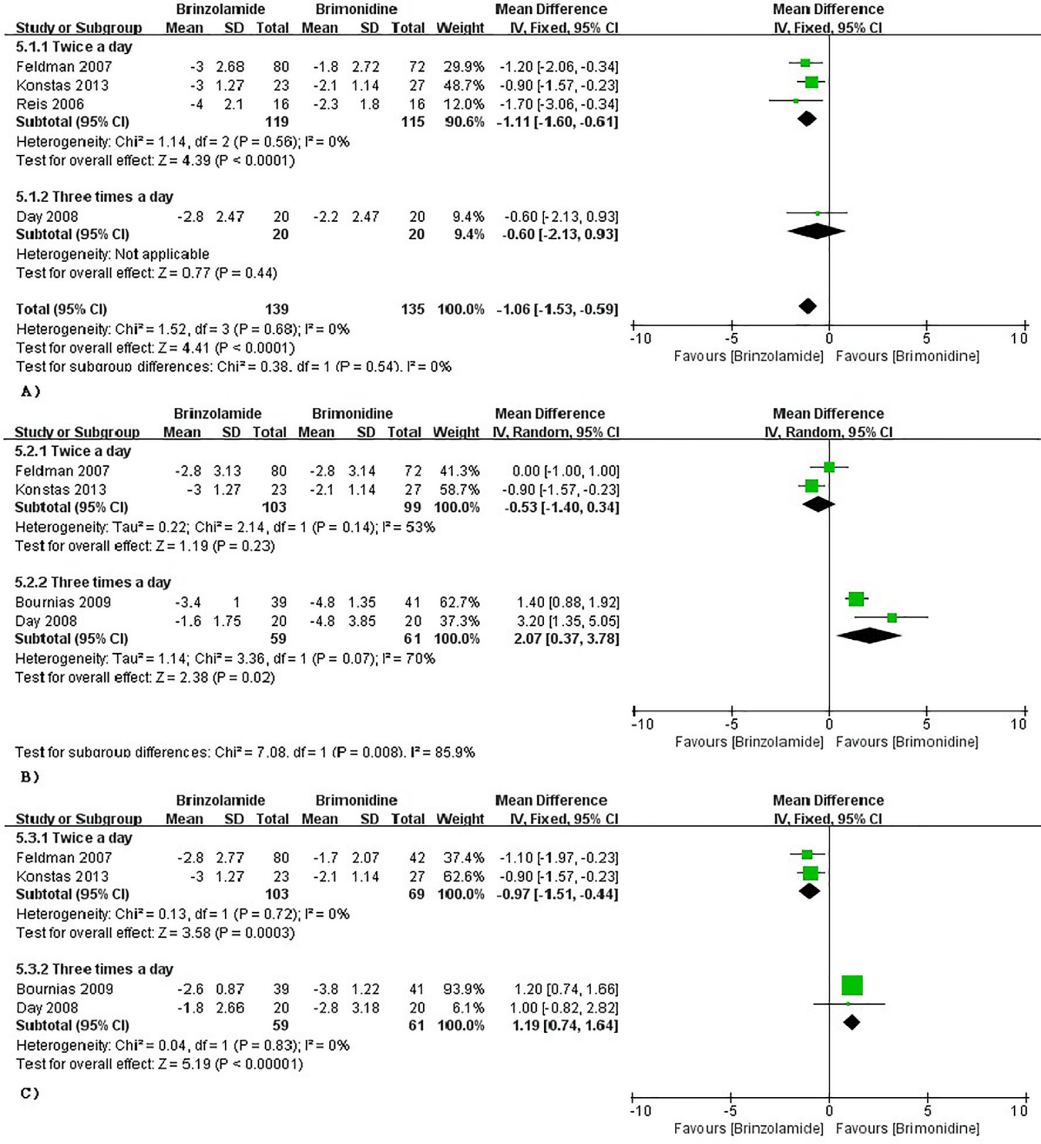
Forest plot for IOP change from baseline (Brinzolamide group vs. Brimonidine group). The plot of change value for IOP at 9 am **(A)**, 12 pm **(B)**, 4 pm **(C)**.

The changes of IOP from baseline between brinzolamide and brimonidine were shown in **Figure 5**. Both drugs significantly decreased IOP as adjunctive therapies to PGAs. At 9 am, a significant reduction of IOP was found in the brinzolamide (b.i.d.) compared to brimonidine (b.i.d.) (WMD −1.11 mmHg, 95%CI [−1.60 to −0.61], *P* < 0.0001, *I* = 0%); however, the difference was not significant in thrice daily subgroup (WMD −0.60 mmHg, 95%CI [−2.13 to –0.96], *P* = 0.44). At 12 am, brinzolamide (b.i.d.) was similar to brimonidine (b.i.d.) in IOP-lowering effect, with a statistically significant heterogeneity (WMD −0.53 mmHg, 95%CI [−1.40 to −0.34], *P* = 0.23, *I* = 53%). When thrice daily, brimonidine led to a greater IOP-lowering effect than brinzolamide, with statistically significant heterogeneity (WMD 2.07 mmHg, 95%CI [0.37 to 3.78], *P* = 0.02, *I* = 70%). In this analysis, we did not perform a sensitivity analysis due to only including two trials in each dose group. At 4 pm, brinzolamide (b.i.d.) had a superior IOP-lowering effect compared with brimonidine (b.i.d.) (WMD −0.97 mmHg, 95%CI [−1.51 to −0.44], *P* = 0.0003, *I* = 0%); conversely, the effect in brinzolamide (t.i.d.) was lower than brimonidine (t.i.d.) (WMD 1.19 mmHg, 95%CI [0.74 to 1.64], *P* < 0.0001, *I* = 0%). With regard to the mean diurnal IOPs at the end, brinzolamide was lower in twice daily subgroup (WMD −1.20 mmHg, 95%CI [−1.31 to 1.08], *P* < 0.00001, *I* = 0%), brimonidine was lower in thrice daily subgroup (WMD 1.41 mmHg, 95%CI [1.02 to 1.80], *P* < 0.00001, *I* = 0%), and the results were also consistent with their IOP changes. There were no publication biases on egger test (*P* ≥ 0.1 for each group; **Supplementary Table 3**). The changes of IOP between brinzolamide and brimonidine could be related to their plasma concentrations and pharmacokinetics (detailed descriptions in the Discussion section).

### Safety Analysis

To obtain a more comprehensive safety profile, we compared the common AEs between brinzolamide and other anti-glaucoma agents, including monotherapies and add-on therapies.

### Blurred Vision and Conjunctival Hyperemia

Blurred vision was one of the most common AEs of brinzolamide. Compared with active comparators (dorzolamide, brimonidine), a greater proportion of patients suffered from blurred vision in brinzolamide (**Table 1**). However, the difference between brinzolamide and timolol was not significant (**Table 1**). For conjunctival hyperemia, the incidence was significantly increased in brinzolamide compared to timolol (**Table 1**); there were no significant differences when comparing brinzolamide with other active comparators (dorzolamide, brimonidine) (**Table 1**).

**Table 1:** Meta-analysis for the efficacy and safety outcomes.

Outcome	Interventions	Studies, (n)	Participants analyzed, n		RR	P value	I**2** **/%**
			Brinzolamide	Comparator	(95% CI)		
Blurred vision	Brinzolamide VS timolol	3	489	323	2.43 [0.95, 5.76]	0.07	0
	Brinzolamide VS dorzolamide	9	988	993	3.24 [1.89, 3.60]	<0.00001	0
	Brinzolamide VS brimonidine	9	1,287	1,260	4.38 [1.36, 14.17]	0.01	63
Ocular discomfort (burning and stinging)	Brinzolamide VS timolol	4	586	418	0.74 [0.27, 1.98]	0.55	52
	Brinzolamide VS dorzolamide	7	711	719	0.21 [0.14, 0.31]	<0.00001	0
	Brinzolamide VS brimonidine	3	90	90	0.85 [0.29, 2.48]	0.76	0
Occurrence of taste abnormality	Brinzolamide VS timolol	3	489	323	6.41 [1.51, 27.16]	0.01	0
	Brinzolamide VS dorzolamide	6	808	806	1.04 [0.69, 1.56]	0.85	0
	Brinzolamide VS brimonidine	9	1,243	1,219	9.61 [5.23, 17.67]	<0.00001	5
Ocular hyperemia	Brinzolamide VS timolol	2	324	250	3.02 [0.48, 19.10]	0.24	0
	Brinzolamide VS dorzolamide	4	545	540	0.45 [0.18, 1.10]	0.08	13
	Brinzolamide VS brimonidine	8	1,053	1,025	0.41 [0.23, 0.73]	0.002	45
Occurrence of conjunctivitis	Brinzolamide VS timolol	2	315	148	0.48 [0.09, 2.70]	0.41	0
	Brinzolamide VS dorzolamide	3	339	336	0.45 [0.15, 1.40]	0.17	13
	Brinzolamide VS brimonidine	4	316	310	0.53 [0.15, 1.93]	0.34	0
Eye pruritus	Brinzolamide VS timolol	3	367	363	1.23 [0.33, 4.52]	0.76	14
	Brinzolamide VS dorzolamide	4	347	350	0.50 [0.17, 1.46]	0.2	59
	Brinzolamide VS brimonidine	4	885	865	0.97 [0.41, 2.26]	0.94	0
Treatment-related adverse events	Brinzolamide VS timolol	3	361	342	1.33 [0.93, 1.91]	0.11	50
	Brinzolamide VS dorzolamide	4	532	543	0.83 [0.49, 1.42]	0.49	79
	Brinzolamide VS brimonidine	5	402	395	1.05 [0.80, 1.39]	0.72	0
Eye pain	Brinzolamide VS timolol	2	324	232	0.88 [0.24, 3.20]	0.85	0
	Brinzolamide VS dorzolamide	2	277	274	0.25 [0.07, 0.88]	0.03	56
	Brinzolamide VS brimonidine	9	1,103	1,075	1.05 [0.61, 1.81]	0.86	0
Foreign body sensation in eyes	Brinzolamide VS timolol	3	421	345	1.56 [0.50, 4.84]	0.44	21
	Brinzolamide VS dorzolamide	4	486	475	0.70 [0.23, 2.16]	0.53	58
	Brinzolamide VS brimonidine	5	507	485	1.29 [0.46, 3.67]	0.63	0
Conjunctival hyperemia	Brinzolamide VS timolol	3	367	363	2.20 [1.14, 4.23]	0.02	0
	Brinzolamide VS dorzolamide	1	98	101	1.03 [0.07, 16.25]	0.98	—
	Brinzolamide VS brimonidine	4	885	865	0.72 [0.31, 1.71]	0.46	0

### Occurrence of Taste Abnormality

Occurrence of taste abnormality was analyzed to have a similar incidence between brinzolamide and dorzolamide (**Table 1**). However, compared with other active comparators (timolol, brimonidine), the reports of occurrence of taste abnormality were significantly higher in brinzolamide (**Table 1**).

### Ocular Discomfort, Eye Pain, and Ocular Hyperemia

Ocular discomfort and eye pain were analyzed to have significantly lower incidences in brinzolamide compared to dorzolamide (**Table 1**); but the differences between brinzolamide and other active (timolol, brimonidine) were not significant (**Table 1**). For ocular hyperemia, the incidence was significantly lower in brinzolamide than brimonidine (**Table 1**); nevertheless, the differences between brinzolamide and other active comparators (timolol, dorzolamide) were not significant (**Table 1**).

### Other Adverse Events

There were no significant differences in the incidence of occurrence of conjunctivitis, eye pruritus, foreign body sensation in eyes, and treatment-related AEs when we compared brinzolamide with active comparators (timolol, dorzolamide, and brimonidine) (all *P* > 0.05; **Table 1**). Besides, no severe AEs were reported in most studies.

## Discussion

In the present systematic review, we assessed 26 RCTs, containing a comparison between brinzolamide and timolol in 7 studies (Silver, 1998; March and Ochsner, 2000; Hollo et al., 2006; Reis et al., 2006; Kaback et al., 2008; Miura et al., 2008; Pfeiffer, 2011), brinzolamide and dorzolamide in 10 studies (Sall, 2000; Michaud and Friren, 2001; Tsukamoto et al., 2005; Bournias and Lai, 2009; Martinez and Sanchez-Salorio, 2009; Manni et al., 2009; Nakamura et al., 2009; Sezgin Akcay et al., 2013; Alcon, 2016; Aihara et al., 2017), and brinzolamide and brimonidine in 9 studies (Feldman et al., 2007; Day and Hollander, 2008; Katz et al., 2013; Konstas et al., 2013; Nguyen et al., 2013; Research, 2013a; Research, 2013b; Whitson et al., 2013; Aung et al., 2014). Patients with POAG or OHT have a common characteristic as elevated IOP, which is closely associated with progression of visual field deterioration. Currently, IOP level control is a primary goal for the treatment of POAG and OHT.

Our analyses found that brinzolamide had similar efficacies to timolol in lowering IOP at three time points (9 am, 12 am, 4 pm) and holding the mean diurnal IOP at the end of treatment duration, as add-on therapies to a PGA, which were not inconsistent with the effects as monotherapies. In a previous meta-analysis, monotherapies were adopted to treat patients with POAG or OHT, and the relative peaks of reduction in IOP were 17% and 27% for brinzolamide and timolol, respectively (van der Valk et al., 2005). Timolol and brinzolamide could reduce formation of AQH; the former decreases blood flow to the iris root–ciliary body while the latter inhibits CAI, and timolol has a stronger effect on the process than brinzolamide (Costagliola et al., 2002; Shoji et al., 2005). PGAs, besides exporting the first-line effect, could enhance the activity of CAI, so brinzolamide is added on PGAs benefiting patients with glaucoma or OHT to achieve further reductions in IOP (Puscas and Coltau, 1995; Miura et al., 2008). However, there is not an interaction between timolol and PGAs. Thus, the efficacy of brinzolamide was similar to timolol as added on PGAs in daytime. Moreover, brinzolamide also lowered the nighttime IOP, although the effect was smaller than during daytime (Liu et al., 2004; Liu et al., 2009). The performance may be explained by the fact that brinzolamide is weaker in reducing the formation of AQH during the nocturnal period than during the diurnal period (Ingram and Brubaker, 1999). In contrast, timolol has no similar effect, because there are normally reductions of endogenous circulating catecholamines in night-time (Topper and Brubaker, 1985; Liu et al., 2004; Liu et al., 2009; Konstas et al., 2016).

As for brinzolamide and dorzolamide, they had same mechanisms that lowered IOP by inhibiting the activity of carbonic anhydrase and enhanced ocular hemodynamic function by retarding the release of intracellular Ca^2+^(Chandra et al., 2016; Dong et al., 2018). Accumulating evidences showed that visual field defect was highly related to the reduction in ocular blood flow (Deokule et al., 2010; Calvo et al., 2012). Therefore, it was reasonable to our results that brinzolamide and dorzolamide had a similar effect in lowering IOP at three time points (9 am, 12 am, 4 pm) and same mean diurnal IOPs at the end of treatment duration, as add-on therapy to a PGA or beta-blocker.

In terms of the comparisons between brinzolamide and brimonidine where some changes had been generated, we implemented subgroup analysis on medication times. At 9 am, brinzolamide was more effective than brimonidine when added to PGAs, and a similar tendency occurred in the brinzolamide group (b.i.d.) at 4 pm. Interestingly, this tendency had reversed in brimonidine (t.i.d.) at 12 am and 4 pm, respectively. However, the difference was not statistically significant comparing brimonidine (b.i.d.) with brimonidine (b.i.d.) at 12 am. In addition, brinzolamide had a lower mean diurnal IOP in twice daily subgroup, but brimonidine had a lower mean diurnal IOP in thrice daily subgroup. The reasons leading to variabilities for therapeutic effect were listed as follows. On the one hand, brinzolamide worked within 30 min and reached a peak after 1–2 h after administration (Silver, 1998). But for brimonidine, the effect was only observed within 1 h, and the peak effect occurred by 2–3 h (Walters, 1996). Nevertheless, the IOP lowering effect of two drugs dropped back to a trough over 10 h after doses (Anderson, 2003; Lusthaus and Goldberg, 2017). On the other hand, the plasma concentrations of two drugs (t.i.d.) were greater than the drugs (b.i.d.), which were more beneficial to the effect of brimonidine than brinzolamide (Fudemberg et al., 2008).

Safety profile of brinzolamide was similar to the other three active comparators when they were used as either monotherapies or adjunctive therapies, but there were some AEs with diverse frequencies. The incidences of blurred vision and taste abnormality were outstanding in brinzolamide. Differing from brinzolamide, ocular discomfort and eye pain were common in dorzolamide; ocular hyperemia was common in brimonidine; timolol led to a low risk of conjunctival hyperemia. All AEs of the four drugs were usually mild and superficial depending on their unique structures; furthermore, brinzolamide with a physiological pH could also ameliorate tolerability and adherence (Silver, 1998).

The present study still had some limitations. First, all articles in our study were published in English; there was a potential risk that we failed to involve some papers that were published in other languages. Second, people with glaucoma and OHT eventually ended up with visual field loss. The patient’s visual field directly reflected the disease progression. However, there was not a precise and accepted visual field detection method at present. Therefore, we evaluated the treatment effect of study drugs by IOP changes from baseline as well as the mean diurnal IOP at the end of treatment duration to restrict the potential bias. Third, due to the lack of data, we did not compare the nocturnal IOP, which also played a dominant role responding to the control level of the disease. It was necessary that more researches were still needed for the available guidance. Finally, there was a lack of cost-effectiveness studies of brinzolamide as an adjunctive therapy.

## Conclusion

This meta-analysis indicated that brinzolamide, as add-on to PGAs or β-blocker, could significantly decrease IOP of people with refractory glaucoma or OHT, and the AEs of brinzolamide were tolerable. Therefore, it could be used as a replacement therapy for patients whose IOP became uncontrollable with a PGA or timolol alone; or as an alternative treatment to patients with contraindications of timolol and brimonidine.

## Author Contributions

YH contributed to devising the topic and writing the manuscript. YL and JZ contributed equally to this work (contributed by writing the manuscript and analyzing the data). XZ and QW contributed to checking the data.

## Conflict of Interest Statement

The authors declare that the research was conducted in the absence of any commercial or financial relationships that could be construed as a potential conflict of interest.
