# Supplementary material for: Efficacy and Safety of Brinzolamide as Add-On to Prostaglandin Analogues or β-Blocker for Glaucoma and Ocular Hypertension: A Systematic Review and Meta-Analysis
Source: Front Pharmacol. 2019 Jun 25;10:679. doi: 10.3389/fphar.2019.00679 (PMC6603202; doi:10.3389/fphar.2019.00679)
Supplement: Supplementary file 1 [file DataSheet_1.docx]

Supplementary Table 1 Characteristics of included studies

| Author （year） | Internentions | Participants | Age | Type of diagnosis | | | Background | Follow-up (weeks) |
| --- | --- | --- | --- | --- | --- | --- | --- | --- |
|  |  |  | (years) | POAG (%) | OHT (%) | Others (%) | therapy |  |
| Sall et al.,  2000 | Brinzolamide 1% tid | N:133 | 63.8 ±12.1 | 70  (21.3) | 217 (66.0) | 7  (2.1) | Monotherapy | 12 |
|  | Dorzolamide 2% tid | N:131 | 64.0±13.3 |  |  |  |  |  |
| Kaback et al., 2008 | Brinzolamide 1% bid | N:173 | NR | 211  (61.0) | 126 (36.4) | 9  (2.6) | Monotherapy | 24 |
|  | Timolol 0.5% bid | N:173 | NR |  |  |  |  |  |
| Silver et al.,  1998 | Brinzolamide 1% bid | N:150 | NR | 300  (58.6) | 206 (40.2) | 6  (1.2) | Monotherapy | 12 |
|  | Brinzolamide 1% tid | N:148 | NR |  |  |  |  |  |
|  | Dorzolamide 2% tid | N:149 | NR |  |  |  |  |  |
|  | Timolol 0.5% bid | N:65 | NR |  |  |  |  |  |
| March et al.,  2000 | Brinzolamide 1% bid | N:153 | 63±11.6 | 136  (59.6) | 82  (36.0) | 10  (4.4) | Monotherapy | 72 |
|  | Timolol 0.5% bid | N:75 | 59.9±13.2 |  |  |  |  |  |
| HOLLÓ et al., 2006 | Brinzolamide 1% bid | N:95 | 63.9±11.7 | 139  (72.4) | 52  (27.1) | 1  (0.5) | Add on to travoprost 0.004% | 12 |
|  | Timolol 0.5% bid | N:97 | 62.2±10.4 |  |  |  |  |  |
| Reis et al.,  2006 | Brinzolamide 1% bid | N:16 | 57.4±10.0 | 46  (88.5) | 6  (11.5) | 0  (0) | Add on to travoprost 0.004% | 4 |
|  | Brimonidine 0.2% bid | N:16 | 54.0±12.0 |  |  |  |  |  |
|  | Timolol 0.5% bid | N:20 | 62.1±10.1 |  |  |  |  |  |
| Miura et al.,  2008 | Brinzolamide 1% bid | N:16 | 66.4±11.2 | 20  (62.5) | 3  (9.4) | 9  (28.1) | Add on to latanoprost | 12 |
|  | Timolol 0.5% bid | N:16 | 63.5±11.5 |  |  |  |  |  |
| Pfeiffer et al., 2011 | Brinzolamide 1% bid | N:96 | 62.7±10.6 | NR | NR | NR | Add on to travoprost 0.004% | 12 |
|  | Timolol 0.5% bid | N:93 | 65.5±13.5 |  |  |  |  |  |
| Aihara et al., 2017 | Brinzolamide1%/timolol 0.5%-FC bid | N:92 | 62.5±11.9 | 154  (81.5) | 35  (19.0) | 0  (0) | Add on to PAG | 8 |
|  | Dorzolamide1%/timolol 0.5%-FC bid | N:97 | 64.1±12.4 |  |  |  |  |  |
| Nakamura et al., 2009 | Brinzolamide 1% bid | N:20 | 65.7±10.2 | 22  (55) | 6  (15) | 12  (30) | Add on to latanoprost 0.005% | 4 |
|  | Dorzolamide 1% bid | N:20 | 65.7±10.2 |  |  |  |  |  |
| Manni et al.,  2009 | Brinzolamide1%/timolol 0.5%-FC bid | N:192 | 65.3 ± 10.9 | 240  (66.7) | 88  (24.4) | 32  (8.9) | Monotherapy | 12 |
|  | Dorzolamide1%/timolol 0.5%-FC bid | N:168 | 64.2 ± 10.2 |  |  |  |  |  |
| Martínez et al., 2009 | Brinzolamide 1% bid | N:76 | 63.7±7.4 | 146  (100) | 0  (0) | 0  (0) | Add on to timolol 0.5% | 240 |
|  | Dorzolamide 2% tid | N:70 | 64.0±8.2 |  |  |  |  |  |
| Tsukamoto et al., 2005 | Brinzolamide 1% bid | N:25 | 61.7±9.0 | 52  (100) | 0  (0) | 0  (0) | Add on to the combi nation  therapy of latanoprost and a beta-blocker | 8 |
|  | Dorzolamide 1% tid | N:27 | 62.1±8.9 |  |  |  |  |  |
| Michaud et al., 2001 | Brinzolamide 1% tid | N:104 | NR | 128  (60.1) | 71  (33.3) | 14  (6.6) | Add on to timolol 0.5% | 12 |
|  | Dorzolamide 1% tid | N:109 | NR |  |  |  |  |  |
| Bournias et al., 2009 | Brinzolamide 1% tid | N:39 | 66.3±8.6 | NR | NR | 0  (0) | Add on to PAG | 16 |
|  | Brimonidine 0.15% tid | N:41 | 64.9±7.6 |  |  |  |  |  |
|  | Dorzolamide 2% tid | N:40 | 65.6±8.0 |  |  |  |  |  |
| Sezgin Akçay et al., 2013 | Brinzolamide1%/timolol 0.5%-FC bid | N:57 | 54.8±8.5 | 40  (69.4) | 12  (21.6) | 5  (9.0) | Monotherapy | 12 |
|  | Dorzolamide1%/timolol 0.5%-FC bid | N:57 | 55.7±5.4 |  |  |  |  |  |
| NCT02325518 2016 | Brinzolamide1%/timolol 0.5%-FC bid | N:98 | 63.1±11.8 | NR | NR | 0  (0) | Add on to PAG | 8 |
|  | Dorzolamide1%/timolol 0.5%-FC bid | N:101 | 64.4±12.3 |  |  |  |  |  |
| Aung et al., 2014 NCT01310777 | Brinzolamide 1% bid | N:191 | 64.1±11.2 | 66  (18.0) | 279 (76.2) | 21  (5.7) | Monotherapy | 24 |
|  | Brimonidine 0.2% bid | N:175 | 64.3±11.6 |  |  |  |  |  |
| Katz et al., 2013  NCT01297517 | Brinzolamide1% tid | N:224 | 65.0±10.0 | 304  (69.1) | 136 (30.9) | 0  (0) | Monotherapy | 12 |
|  | Brimonidine 0.2% tid | N:216 | 64.3±10.8 |  |  |  |  |  |
| NCT01297920 2013 | Brinzolamide1% tid | N:234 | NR | NR | NR | 0  (0) | Monotherapy | 12 |
|  | Brimonidine 0.2% tid | N:235 | NR |  |  |  |  |  |
| Nguyen et al., 2013 | Brinzolamide1% tid | N:229 | 64.2 ±10.3 | 344  (74.6) | 117 (25.4cs) | 0  (0) | Monotherapy | 12 |
|  | Brimonidine 0.2% tid | N:232 | 64.9 ±10.5 |  |  |  |  |  |
| Day et al., 2008 NCT00440141 | Brinzolamide1% tid | N:20 | 57.5 ± 14.1 | 3  (7.5) | 32  (80) | 5  (12.5) | Add on to latanoprost 0.005% | 12 |
|  | Brimonidine 0.1% tid | N:20 | 60.0 ± 11.6 |  |  |  |  |  |
| Feldman et al., 2007 | Brinzolamide 1% bid | N:84 | 64.00±10.25 | 109  (66.9) | 48  (29.4) | 6  (3.7) | Add on to travoprost 0.004% | 12 |
|  | Brimonidine 0.15% bid | N:79 | 62.91±9.34 |  |  |  |  |  |
| NCT00961649 2013 | Brinzolamide1% tid | N:44 | NR | NR | NR | 0  (0) | Monotherapy | 6 |
|  | Brimonidine 0.2% tid | N:41 | NR |  |  |  |  |  |
| Whitson et al., 2013 | Brinzolamide 1% tid | N:229 | 64.2±10.3 | 342  (74.2) | 117 (25.4) | 0  (0) | Monotherapy | 24 |
|  | Brimonidine 0.2% tid | N:232 | 64.9±10.5 |  |  |  |  |  |
| Konstas et al., 2013 | Brinzolamide1%/timolol 0.5%-FC bid | N:50 | 64.2± 9.8 | 33  (66.0) | 0 (0) | 17 (34.0) | Add on to travoprost 0.004% | 12 |
|  | Brimonidine0.2%/timolol 0.5%-FC bid | N:50 | 64.2± 9.8 |  |  |  |  |  |

Supplementary Table 2 Risk of bias included trials

| Study | Random sequence generation | Allocation concealment | Blinding | Incomplete outcome data | Selective  outcome reporting | ITT | Lost to follow-up |
| --- | --- | --- | --- | --- | --- | --- | --- |
| Sall et al., 2000 | U | L | L | L | L | L | L |
| Kaback et al., 2008 | U | L | L | L | L | L | L |
| Silver et al.,1998 | L | L | L | L | L | H | L |
| March et al.2000 | U | L | L | L | L | L | L |
| HOLLÓ et al., 2006 | U | L | L | L | L | L | L |
| Reis et al., 2006 | U | U | L | L | L | H | L |
| Miura et al., 2008 | L | L | U | L | L | H | L |
| Pfeiffer et al., 2011 | U | L | L | L | L | L | L |
| Aihara et al., 2017 | U | U | L | L | L | H | L |
| Nakamura et al., 2009 | U | U | U | L | L | H | L |
| Manni et al., 2009 | L | U | L | L | L | L | L |
| Martínez et al.2009 | U | U | L | L | L | L | L |
| Tsukamoto et al., 2005 | U | U | H | L | L | H | L |
| Michaud et al., 2001 | U | U | L | L | L | L | L |
| Bournias et al., 2009 | L | L | L | L | L | L | L |
| Sezgin Akçay et al.2013 | U | U | L | L | L | H | L |
| NCT02325518 2016 | U | U | L | L | L | H | L |
| Aung et al., 2014 NCT01310777 | L | L | L | L | L | L | L |
| Katz et al.2013  NCT01297517 | L | L | L | L | L | L | L |
| NCT01297920 2013 | U | U | L | L | L | L | L |
| Nguyen et al.2013 | U | U | L | L | L | L | L |
| Day et al., 2008  NCT00440141 | U | L | L | L | L | H | L |
| Feldman et al., 2007 | U | L | L | L | L | L | L |
| NCT00961649 2013 | U | U | L | L | L | L | L |
| Whitson et al.2013 | U | U | L | L | L | L | L |
| Konstas et al., 2013 | U | U | L | L | L | H | L |

U=Unclear risk of bias, L=Low risk of bias, H= High risk of bias, ITT=Intent to treat

Supplementary Table 3 Egger test outcomes

| Outcome & Interventions | Time-point of measurement | Studies （n） | [95% CI] | P value |
| --- | --- | --- | --- | --- |
| IOP change from baseline  (Brinzolamide VS Timbol) | 9 am | 3 | [-65.13, 59.95] | 0.692 |
|  | 12 am | 4 | [-48.91, 54.95] | 0.595 |
|  | 4 pm | 2 | / | / |
| IOP change from baseline  (Brinzolamide VS Dorzolamide) | 9 am | 5 | [-2.51, 1.58] | 0.522 |
|  | 12 am | 6 | [-0.54, 0.49] | 0.917 |
|  | 4 pm | 4 | [-1.05, 1.38] | 0.619 |
| IOP change from baseline  (Brinzolamide VS Brimonidine) | 9 am | 4 | [-6.26, 5.21] | 0.731 |
|  | 12 am | 4 | [-23.05, 24.61] | 0.901 |
|  | 4 pm | 4 | [-24.30, 18.08] | 0.592 |
| The mean diurnal IOPs  (Brinzolamide VS Timbol) | / | 5 | [-33.96, 32.51] | 0.827 |
| The mean diurnal IOPs  (Brinzolamide VS Dorzolamide) | / | 5 | [-2.03, 2.09] | 0.966 |
| The mean diurnal IOPs  (Brinzolamide VS Brimonidine) | / | 5 | [-61.52, 90.90] | 0.583 |
